# Trayectoria tecnológica y productiva de la industria de vacunas humanas en México: perspectivas post Covid-19

**DOI:** 10.15446/rsap.V25n5.110791

**Published:** 2023-09-01

**Authors:** María del Pilar M. Pérez-Hernández, Luis M. Castillo-Chávez

**Affiliations:** 1 MP: Lic. Economía. Ph. D. Economía y Gestión de la Innovación y Política Tecnológica. M. Sc. Economía y Gestión del Cambio Tecnológico. Centro de Investigaciones Económicas, Administrativas y Sociales (CIECAS), Instituto Politécnico Nacional (IPN). Ciudad de México, México. mpperez@ipn.mx Instituto Politécnico Nacional Instituto Politécnico Nacional Ciudad de México Mexico mpperez@ipn.mx; 2 LC: MD. Ph.D.; M.Sc. Ciencias Biológicas. Centro de Investigaciones Económicas, Administrativas y Sociales (CIECAS), Instituto Politécnico Nacional (IPN). Ciudad de México, México. luis.castillo.chavez@gmail.com Instituto Politécnico Nacional Instituto Politécnico Nacional Ciudad de México Mexico

**Keywords:** Vacunas, México, industria farmacéutica, desarrollo tecnológico *(fuente: DeCS, BIREME)*, Vaccines, Mexico, drug industry, sustainable development *(source: MeSH, NLM)*

## Abstract

Las vacunas han transformado la salud a escala global. La perturbación causada por la pandemia de covid-19 reveló la fragilidad de los sistemas de salud en todo el mundo. Aquellos países con capacidades en el desarrollo y la fabricación de vacunas impulsaron el incremento del financiamiento para el desarrollo de posibles vacunas. México, otrora líder en producción de vacunas a escala mundial, pasó décadas de abandono y escasa capacidad de respuesta para atender a las necesidades que trajo consigo la pandemia. Este trabajo analiza la evolución de las capacidades de la industria productora de vacunas en México. La metodología hace una revisión crítica de la literatura con respecto a la evolución de la trayectoria productiva y tecnológica de la industria de las vacunas. Los hallazgos apuntan a que la industria de vacunas en México, derivado del deterioro de la inversión en el sector, la falta de flexibilidad organizacional de la empresa pública productora de vacunas y la ausencia de políticas públicas experimentó la pérdida de su capacidad para generar autonomía en la fabricación de vacunas, por lo que es imperioso revisar la necesidad de disponer de capacidades locales para la fabricación de vacunas como un área estratégica del desarrollo de la nación.

## Abstract (en)

Vaccines have transformed health globally. The disruption caused by the Covid-19 pan-demic revealed the fragility of healthcare systems around the world. Those countries with capacities in the development and manufacture of vaccines promoted the increase in financing for the development of possible vaccines. In the case of Mexico, once the world leader in vaccine production, it exhibited decades of neglect and little response capacity to meet the needs brought about by the pandemic. This paper analyzes the evolution of the capacities of the vaccine production industry in Mexico. The methodology makes a critical review of the literature regarding the evolution of the productive and technological trajectory of the vaccine industry. The findings point to the fact that the vaccine industry in Mexico, derived from the deterioration of investment in the sector, the lack of organizational flexibility of the public company that produces vaccines, and the absence of public policies, caused the loss of the capacity to generate autonomy in the vaccine manufacturing, it is therefore imperative to review the need to have local capacities for vaccine manufacturing as a strategic area of development for the nation.

Las vacunas han transformado el panorama de la salud a escala global. De acuerdo con la Organización Mundial de la Salud (OMS), cada año se evitan entre dos y tres millones de muertes gracias a ellas en el mundo. A escala global, se salvan 178,28 millones de años de vida anuales y 43,57 millones de años de vida ajustados por discapacidad (AVAD), medida que expresa el tiempo que una persona vive con discapacidad como consecuencia de la erradicación y el control de enfermedades como la viruela, el polio, el sarampión o el tétanos 1,2.

Las vacunas son un campo de creciente desarrollo, pero hay momentos en la humanidad en los cuales su papel es decisivo, como, por ejemplo, cuando el 31 de diciembre del 2019 se reportaron 41 casos de neumonía de origen incierto en China, en marzo del 2020 la OMS declaró que era una pandemia, y hasta el 1 de junio del 2023 se tienen registrados más de 767 millones de casos a escala mundial, con más de 6,9 millones de muertes 3. La emergencia obligó a los gobiernos a restringir la libertad de movimientos de las empresas y los individuos, lo cual cambió por completo las prioridades de la industria farmacéutica. Se han impulsado diferentes estrategias para encontrar mecanismos y opciones de vacunación; la gran apuesta para frenar la pandemia es alcanzar tasas suficientes de vacunación 1.

La pandemia de covid-19 generó un mayor interés por comprender el papel de la transferencia de tecnología como medio para aumentar la producción mundial de vacunas. Para responder a la demanda sin precedente de vacunas, el desarrollo y la producción de la vacuna covid-19 requirió que hubiese transferencia tecnológica entre países desarrolladores y empresas farmacéuticas de todo el mundo, lo que ha sido objeto de un debate 4.

Las inversiones continuas en innovación de vacunas no se traducen suficientemente en la entrada de productores al mercado de nuevas vacunas. Esta paradoja de la innovación se debe en cierta medida a que las partes interesadas carecen de una comprensión completa sobre el desarrollo de vacunas y las dificultades de colaboración entre la amplia variedad de grupos de interés involucrados (5,6). Muchos países cuando tienen problemas económicos recortan el gasto en salud; sin embargo, las pandemias son un recordatorio para todos del papel estratégico de este sector para el desarrollo de cualquier país. México, otrora líder en la fabricación de vacunas, desde la década de 1990 redujo la inversión para la atención de estas necesidades, por lo que la pandemia de covid-19 tomó al país en una situación vulnerable que requiere ser examinada 7.

Este trabajo se enmarca en los estudios de la economía de la innovación, los cuales, por medio de los constructos analíticos como trayectoria tecnológica y los sistemas sectoriales de innovación, permiten caracterizar la trayectoria productiva y tecnológica a partir del grado de innovación o madurez tecnológica de la industria de vacunas humanas en México. El trabajo se divide en tres secciones, en la cuales se analizan los desafíos de la industria de vacunas en el mundo, después se caracteriza la industria productora de vacunas en México, para finalmente discutir y analizar las perspectivas futuras más allá de la pandemia.

## Trayectoria tecnológica de la industria de vacunas en el mundo

La trayectoria tecnológica considera procesos de acumulación de conocimientos, de capacidades y de recursos, por lo que los esfuerzos pasados repercutirán en los resultados futuros (path dependence), por lo cual la evolución de las empresas está definida por su trayectoria tecnológica 8. El sistema sectorial de innovación permite comprender el papel de la coevolución interactiva de diversos factores tecnológicos e institucionales en el crecimiento de la productividad de los distintos sectores económicos 9. La industria de las vacunas ha coevolucionado, pues su desarrollo y su producción involucran considerables inversiones, incluidos diferentes tipos de capacidades (científicas, tecnológicas y de innovación). Aun así, nada garantiza el éxito final del producto, por lo que se considera que es una industria basada en el conocimiento.

El proceso de vacunación requiere incentivos apropiados para fomentar el descubrimiento oportuno y el desarrollo innovador, eficaz, seguro y asequible de productos, como también financiamiento efectivo y programas de entrega, líderes científicos creíbles para proporcionar recomendaciones de política pública basadas en evidencia y tranquilizar al público sobre el valor de las vacunas. Los avances en las políticas de salud también han mejorado los efectos de la vacuna a escala global 10.

**Figura 1:**
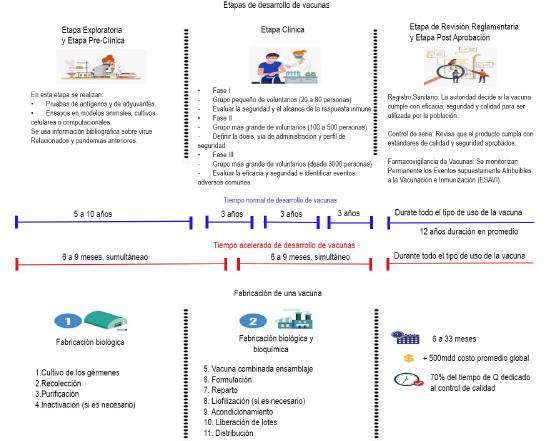
Ciclo del desarrollo de vacunas humanas Etapas de desarrollo de vacunas

El desarrollo y la fabricación de una vacuna es un proceso largo y complejo, con varias etapas rigurosamente controladas de inicio a fin, pues tienen que cumplir los máximos estándares de calidad y seguridad, durante toda la cadena de producción. Ejemplo de ello es que más del 70 % del tiempo de elaboración de una vacuna se invierte en controles de calidad (análisis de pureza, eficacia, control microbiológico e inocuidad), que se realizan sistemáticamente sobre cada lote de vacuna 10,11. Esto constituye una de las grandes barreras de entrada, pues se requieren 6 a 22 meses para producir una vacuna y hasta 15 años para lograr los controles de las fases clínicas y que se autorice su comercialización (Figura 1) 11-13.

Fuente: tomado de CENDHY 33.

Las vacunas se han hecho más accesibles en países emergentes, pero la vigilancia de la seguridad y la supervisión regulatoria no siempre son óptimas en estos países, por lo cual las vacunas falsificadas siguen siendo una amenaza. La velocidad y el alcance de la información en las plataformas de redes sociales han creado oportunidades sin precedentes para que los usuarios amplifiquen la desinformación y fomenten los recelos al proceso de vacunación 10.

Esta dinámica de acelerado cambio tecnológico en la biotecnología ha redefinido el proceso de innovación de la industria farmacéutica, lo cual ha profundizado las diferencias de capacidades entre los países. Así, mientras los países industrializados incentivan el esfuerzo innovador con políticas públicas, en los países de industrialización tardía se promueven capacidades adaptativas y tecnológicas mediante la trasferencia de tecnología tanto activa como pasiva 14.

El mercado global de vacunas está en constante crecimiento, a una tasa anual del 16,2 %>; para el 2021 registró 48 billones de dólares en ventas. Las innovaciones en vacunas han sido desarrollas por grandes empresas farmacéuticas; es preciso reconocer los esfuerzos de los países de industrialización tardía por producir vacunas necesarias para sus respectivos países 15. La industria farmacéutica a escala mundial está concentrada en Estados Unidos, Europa, Japón, China y Corea del Sur. Por su elevada intensidad tecnológica, la competitividad de la industria de las vacunas depende de la habilidad de las empresas para desarrollar productos novedosos, en tanto que la actividad innovadora está asociada a la capacidad para financiar la investigación y el desarrollo (I+D). La competitividad de los países en este sector depende de las políticas públicas para fomentar la actividad innovadora 14.

**Figura 2:**
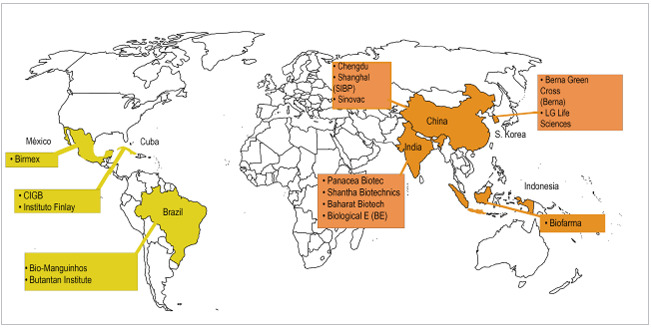
Países emergentes en el desarrollo de vacunas

La Alianza Internacional de Manufacturas de Vacunas de Países en Desarrollo (DCVMN, por sus siglas en inglés) es una estrategia de los países de industrialización, de acuerdo con la cual organizaciones públicas y privadas (conformadas por 37 manufactureras de 14 países) trabajan para producir y proveer vacunas de alta calidad a precios accesibles para proteger a la población mundial de enfermedades desconocidas y emergentes (Figura 2) 16-18.

La pandemia de covid-19 generó una atención renovada a la transferencia de tecnología. Así, los cambios recientes en la normatividad de la propiedad intelectual (PI), que requiere la extensión de patentes a productos farmacéuticos, generan preocupaciones sobre cómo la PI afectará el acceso global a medicamentos esenciales 4.

Un hito importante de la pandemia de covid-19 ha girado en torno a la inédita rapidez con que se obtuvieron las vacunas. Cuando la producción depende de la participación del creador de la tecnología, para ayudar a dominar los procesos de producción y satisfacer la regulación, la ausencia o eliminación de PI es poco probable que aumente la oferta mundial. Lo que se necesita es una PI aditiva, es decir, la transferencia de tecnología del generador a los socios 4.

La transferencia de tecnología durante la pandemia de covid-19 presentó nuevos desafíos. Debido a que muchas de las vacunas para covid-19 se basan en nueva tecnología genética recombinante, las plataformas disponibles que usaban dicha tecnología tenían un limitado uso en humanos; por ello, el diseño y la creación de la vacuna para covid-19 para humanos requirió prácticamente una construcción desde cero. Por esta razón, varias fases que suelen ser secuenciales se realizaron simultáneamente y sin concluirse para acelerar el proceso de obtención de la vacuna. Por ende, para satisfacer la demanda masiva y urgente creada por la pandemia, varios de los desarrolladores establecieron redes globales de proveedores y productores que requieren la transferencia de tecnología y know-how de fabricación para nuevos socios. Las cadenas de suministro de vacunas también sufrieron graves interrupciones en el 2021, debido a la enorme demanda de insumos y a las restricciones a la exportación que algunos países impusieron 4.

## Vacunas en México una historia con luces y sombras

En México los antecedentes de la producción de vacunas se remontan a la Colonia, con los desarrollos de Balmis, la adopción rápida de los avances en la microbiología que se difundían en forma altruista por todo el mundo, y la posterior formación en el extranjero de capital humano en los centros de investigación de excelencia de Europa y Estados Unidos. La producción de vacunas inició en 1939 en el Instituto Nacional de Higiene (INH), y hacia 1970 México aparecía entre los primeros siete lugares del mundo como productor del Programa Ampliado de Inmunizaciones (PAI) de la Organización Panamericana de la Salud (OPS); en este periodo se producía el 90 % de las vacunas requeridas. Los esfuerzos en la vacunación y la producción de vacunas fueron continuados por los diferentes gobiernos, lo que le permitió al país incorporar rápidamente avances hechos en otras latitudes 19.

Las vacunas hicieron que en México, entre 1990 y el 2010, luego de la puesta en marcha del Sistema Nacional de Vacunación, la mortalidad infantil bajara de 47,1 a 16,8 muertes por 1 000 nacimientos. El gasto per cápita en salud en el 2010 fue de 894,2 dólares y en el 2020 pasó a 1 197,7 dólares, lo que representa el 6 % del producto interno bruto (PIB) nacional 1.

La reestructuración económico-productiva de mediados de la década de 1990, junto a los procesos de modernización y apertura comercial y el adelgazamiento del Estado 7,20, trajeron consigo un viraje en las políticas públicas. El progreso social cedió paso a la modernización industrial y a la competitividad 21.

Lo anterior ocasionó la reducción de la capacidad técnica para la producción de antígenos (se redujo el gasto y se desmantelaron los centros de I+D de vacunas, a pesar de producirse el 90 % de las vacunas requeridas); se fusionaron y desaparecieron instituciones, hasta conformarse la empresa paraestatal Birmex en 1999; y se incrementó la importación de productos biofarmacéuticos, con lo que México dejó de ser autosuficiente en la producción de vacunas 7,22.

La autosuficiencia en la producción de vacunas en México tiene un difícil camino, pues desde 1998 no se producen suficientes vacunas, ni siquiera las contempladas en el esquema nacional de vacunación. Birmex compra vacunas a las farmacéuticas internacionales y tiene acuerdos con Glaxo, Merck y Sanofi 7,20.

Se puede afirmar que en la trayectoria tecnológica de México, desde la década de 1990 se fue abriendo una brecha en las capacidades tecnológicas y productivas con respecto a los avances a escala mundial. Con la pandemia por influenza N1H1 se hicieron esfuerzos para producir vacunas (en alianza con Sanofi). En el caso del covid-19, ante la falta de capacidades para desarrollar una vacuna y el hecho de tener acceso a las vacunas que se perfilaban para combatir la enfermedad, México participó en el mecanismo Covax para reservar la compra de los diferentes prototipos de vacunas, e incluso ha participado en el envase de estas para su distribución (mediante Birmex), de las vacunas Astra-Zeneca y CanSino 23.

La pandemia modificó el papel de Birmex, qué desincorporó sus actividades, y la producción de vacunas se ha transferido en la actualidad al INH. En el caso de la vacuna CanSino, para México se acordó que esta se fabricaría por medio de la empresa Drugmex, en lugar de las instalaciones del sector público 24.

En cuanto a la fabricación de la vacuna contra el virus SARS-CoV-2, se impulsó el proyecto Patria, desarrollado por la empresa Arvimex con tecnología de la ICAHN School of Medicine at Mount Sinai y la Universidad de Texas (ambas de los Estados Unidos). La Universidad Nacional Autónoma de México (UNAM) y el Instituto Nacional de Enfermedades Respiratorias (INER) hicieron las pruebas analíticas de la vacuna y la fase clínica, la cual se desarrolló solamente en el Hospital Médica Sur 25. Iniciativas como la vacuna Patria no han estado exentas de debates y controversias, a la luz de su excesivo costo, la subrogación del proceso de laboratorio, la escasa capacidad de producción, los considerables beneficios de las grandes empresas farmacéuticas, la demora en la conclusión de los proyectos, dudas sobre su seguridad y la escasa articulación con las capacidades científicas y tecnológicas del país 26,27.

La creación y producción de vacunas requiere inversiones considerables, como por ejemplo la existencia de laboratorios biológicos BSL-3 y BSL4, que son los únicos que cuentan con las características para el manejo, la vigilancia epidemiológica y la identificación de microorganismos asociados con enfermedades humanas letales; es en este tipo de laboratorios donde se lleva a cabo el manejo que se requiere para la creación de vacunas 28. Los laboratorios BSL-3 y BSL-4 solo representan la capacidad tecnológica para el diseño de las vacunas, pero no la infraestructura o los recursos para la aplicación de las fases clínicas y la fabricación de las vacunas de manera masiva. La disponibilidad de la tal infraestructura en México es escasa pues solo hay cuatro laboratorios nivel BSL-3, dos en Ciudad de México (Instituto de Diagnóstico y Referencia Epidemiológicos (INDRE) y UNAM), uno en Jalisco (Centro de Investigación y Asistencia en Tecnología y Diseño del Estado de Jalisco [CIATEJ]) y otro en Nuevo León (Universidad Autónoma de Nuevo León [UNAL]). Hasta el momento no se tiene registro de laboratorios biológicos grado BSL-4. Es menester mencionar que los laboratorios BSL-3, que permiten la fabricación de ciertas vacunas a nivel de diseño, no cuentan con la infraestructura para las fases clínicas o la fabricación 28-30.

Recientemente se ha impulsado el desarrollo de capacidades científicas y tecnológicas con la creación del Laboratorio Nacional de Vacunología y un posgrado asociado a este en el 2022. Con respecto a las capacidades científicas y tecnológicas, en México, luego de revisar las patentes registradas en Patentscope, se observa que de las 19 patentes otorgadas del 2010 a la fecha 31, las empresas nacionales contribuyen con el 26,3%; los centros públicos de investigación (CPI) aportan el 21,05 %, lo mismo que los independientes; los institutos nacionales de salud (INS) aportan el 15,78 %; las empresas extranjeras el 10,52 % y las instituciones de educación superior (IES) el 5,26 %. Estas cifras, contrastadas con la infraestructura disponible para el escalamiento de las vacunas abre preguntas del grado de desarrollo de las invenciones protegidos que deberán indagarse. En términos de su aportación regional, en la Ciudad de México, Jalisco y Puebla se encuentran radicados los inventores.

En cuanto a la producción de artículos asociados a vacunas en México, se encontró que entre el 2017 y el 2023 se publicaron 24 artículos en Pubmed, de los cuales 11 tratan sobe la vacuna de covid-19 y sus efectos clínicos, mientras que 13 se relacionan con vacunas de diferentes tipos. Estas publicaciones, sin embargo, no corresponden al diseño de vacunas, sino solamente al estudio de sus efectos en la población 32.

De los artículos indexados en la Web of Science sobre vacunas, dados a conocer entre el 2017 y el 2023, se identificaron 433, los cuales la principal enfermedad que tratan es el covid-19 (17,78 %, 77 artículos), mientras que el resto se ocupa de diferentes patologías. Estos artículos abordan tanto el uso de las vacunas como su efecto en los pacientes y la experimentación de pruebas en animales, entre otros aspectos, pero solo 4 se enfocan en el desarrollo y el diseño de vacunas.

Con referencia a publicaciones sobre la vacuna Patria, solo se identificó una publicación en la Web of Science, publicada en el 2021, que corresponde a la investigación realizada por Avimex, el Instituto ICAHN y el Instituto Mexicano del Seguro Social (IMSS), los cuales, como se mencionó, son los encargados del desarrollo de la vacuna mexicana para el covid-19.

Con la información disponible, se puede observar que no existe claridad en cuanto a cómo se eslabonan las capacidades científicas y tecnológicas de las IES y los CPI con los entes reguladores y normativos, las empresas y otros actores del sistema de innovación; lo que se observa es una fragmentación y una estrategia reactiva que preserva la dependencia tecnológica.

La decisión política de apoyar las actividades de desarrollo y producción de vacunas debe traducirse en la asignación de presupuestos específicos para las iniciativas de desarrollo tecnológico de vacunas e inversiones en la modernización de laboratorios y equipos, así como cambios institucionales y organizacionales para proveer mecanismos de gestión administrativa y tecnológica, de acuerdo con las características y los desafíos que el propio sector tiene.

Es de vital importancia desarrollar una estrategia regional de cooperación técnica y comercial entre los países de América Latina en la cual los procesos de transferencia acompañen a un sistema que potencie las capacidades tecnológicas existentes y se posibilite la producción de otras vacunas. También resulta importante desarrollar proyectos multiinstitucionales que incluyan a los grupos de investigación existentes en la región, lo que permitiría organizar un programa regional de desarrollo de vacunas, de manera que se reduzca el periodo de generación de una nueva vacuna y los costos de inversión. La ventana de oportunidad tecnológica que abrió la pandemia para acelerar los procesos de transferencia de tecnología resulta determinante para el desarrollo ulterior del sistema de innovación en vacunas. El cambio tecnológico ofrece una manera de contrarrestar esa incertidumbre y gestionar de una mejor manera los riegos. La dinámica social, productiva y económica que afectó durante dos años debe ser un recordatorio de la importancia y la necesidad de estar mejor preparados y aprender la lección.

El desarrollo de capacidades es un proceso arduo y continuo que hace posible a los países de menor desarrollo tecnológico reducir las brechas con los productores del conocimiento ♦

## References

[1] Asociación Mexicana de Industrias de Investigación Farmacéutica (AMIIF) (2021). 70 años. Una historia de innovación.

[2] Bloom DE (2010). The value of vaccination. Hot topics in infection and immunity in children VII.

[3] World Health Organization (WHO) (2023). WHO coronavirus (covid-19) dash-board.

[4] da Fonseca EM, Shadlen KC, de Moraes Achcar H (2023). Vaccine technology transfer in a global health crisis: Actors, capabilities, and institutions. Res Policy.

[5] Maxmen A (2021). he fight to manufacture covid vaccines in lower-income countries. Nature.

[6] McMahon A (2021). Global equitable access to vaccines, medicines and diagnostics for covid-19: The role of patents as private governance. J Med Ethics.

[7] Cuevas Mercado NA, Amaro Rosales M (2023). Aspectos socioeconómicos e institucionales de la biotecnología en México.

[8] Villazul JJ (2004). Trayectoria tecnológica y ciclo de vida de las empresas: una interpretación metodológica acerca del rumbo de la innovación. Contaduría y Administración.

[9] McKelvey M, Orsenigo L, Pammolli F (2004). Pharmaceuticals analyzed through the lens of a sectoral innovation system. Sectoral systems of innovation.

[10] Gerberding JL, Haynes BF (2021). Vaccine innovations - past and future. N Engl J Med.

[11] Alsina P (2017). Las vacunas, su proceso de fabricación y otras causas del desabastecimiento.

[12] Douglas RG, Samant VB (2018). The vaccine industry. Plotkin's Vaccines.

[13] Kaddar M, Milstien J, Schmitt S (2014). Impact of BRICS? Investment in vaccine development on the global vaccine market. Bull World Health Organ.

[14] Guzmán A, Pérez MA (2020). Extensión de la pandemia covid-19 frente al acceso a la vacuna y las capacidades tecnológicas y de innovación del sector biofarmacéutico de México. Economía: Teoría y Práctica.

[15] Statista (2023). Covid 19 Vaccines-Worldwide.

[16] Pagliusi S, Leite LC, Datla M, Makhoana M, Gao Y, Suhardono M (2013). Developing countries vaccine manufacturers network: doing good by making high-quality vaccines affordable for all. Vaccine.

[17] Lee AJ, Landau R (2017). Aortocaval compression syndrome: time to revisit certain dogmas. Anest Analg.

[18] Hayman B, Suri RK, Downham M (2022). Sustainable vaccine manufacturing in low-and middle-income countries. Vaccine.

[19] Santos JI (2014). La vacunación en México en el marco de las "décadas de las vacunas": logros y desafíos. Gac Med Mex.

[20] Tamez S, Eibenschutz C, Zafra X, Ramírez R (2016). articulación público-privada en la producción de vacunas en México. Saúde em Debate.

[21] Casas R, Dettmer J (2007). Construyendo un paradigma de política científico tecnológica para México. Educación, ciencia, tecnología y competitividad México D. F.: Miguel Ángel Porrúa.

[22] Santos JI (2002). El Programa Nacional de Vacunación: orgullo de México. Rev Fac Med UNAM.

[23] Gobierno de México (2021). Recibe México dos millones de vacunas contra COVID-19 en dos embarques.

[24] Malacalza B, Fagaburu D (2022). ¿Empatía o cálculo? Un análisis crítico de la geopolítica de las vacunas en América Latina. Foro Internacional.

[25] Avimex (2021). Avimex® anuncia el desarrollo de Patria: vacuna mexicana contra SARS-CoV-2.

[26] Ramírez Coronel M (2023). La vacuna Patria y sus pendientes.

[27] Suarez K (2022). Patria", el sueño de una vacuna mexicana contra la covid-19, se aleja por falta de recursos y voluntarios.

[28] Villegas HHL, Núñez NVA, Padilla CR (2007). Laboratorios de bioseguridad nivel 3 y 4. Rev Mex Patol Clin.

[29] Centro Nacional de Biotecnología (CNB) (2017). Manual de Seguridad e Higiene en los laboratorios.

[30] World Health Organization (WHO) (2010). Responsible life sciences research for global health security: a guidance document.

[31] World Intellectual Property Organization (WIPO) (2023). Patentscope: présentation globale.

[32] ("México"[cy]) AND (vaccine[Title]) (2023). National Library of Medicine.

[33] Advance Center for Chronic Diseases (ACCDIS) (2020). CENDHY - vacuna covid-19: no es una carrera de velocidad.

[34] Kaddar M (2013). Global vaccine market features and trends.

